# Retraction: An effective hotspot mitigation system for Wireless Sensor Networks using hybridized prairie dog with Genetic Algorithm

**DOI:** 10.1371/journal.pone.0362163

**Published:** 2026-11-05

**Authors:** The PLOS One Editors

The *PLOS One* Editors retract this article [1] due to concerns about potential manipulation of the publication process. These concerns call into question the validity and provenance of the reported results. We regret that the issues were not identified prior to the article’s publication.

Additionally, the following concerns were noted:

- Reference 3 was retracted after [1] was submitted and before [1] was published.
- References 24 and 28 were retracted after [1] was published.

The author either did not respond directly or could not be reached.
